# Prognostic value of systemic inflammation score in patients with hepatocellular carcinoma after hepatectomy

**DOI:** 10.18632/oncotarget.18121

**Published:** 2017-05-24

**Authors:** Shiming Shi, Qing Chen, Luxi Ye, Dan Yin, Xuedong Li, Zhi Dai, Jian He

**Affiliations:** ^1^ Department of Radiation Oncology, Zhongshan Hospital, Fudan University, Shanghai, P.R. China; ^2^ Liver Cancer Institute, Zhongshan Hospital, Fudan University, Shanghai, P.R. China; ^3^ Department of General Surgery, Zhongshan Hospital South, Fudan University, Shanghai Public Health Clinical Center, Fudan University, Shanghai, P.R. China; ^4^ Institute of Biomedical Sciences, Fudan University, Shanghai, P.R. China

**Keywords:** hepatocellular carcinoma, blood lymphocyte-to-monocyte ratio, gamma-glutamyltransferase, systemic inflammation score, hepatectomy

## Abstract

Inflammation plays an important role in cancer progression. In this study, we aimed to investigate the prognostic value of the systemic inflammatory biomarkers in hepatocellular carcinoma (HCC) patients undergoing curative resection. Data from 271 HCC patients who underwent curative resection in Zhongshan Hospital between 2008 and 2011 were included. Kaplan-Meier survival analysis showed that gamma-glutamyltransferase (GGT) and lymphocyte-to-monocyte ratio (LMR) were significantly associated with overall survival(OS) and time to recurrence(TTR). We created a systemic inflammation score (SIS) basing on preoperative serum GGT and LMR. Low SIS was also significantly associated with increased OS and TTR. Univariate and multivariate analyses revealed the LMR, GGT and SIS were independent predictors for OS and TTR. The predictive ability of the SIS, as assessed by area under the receiver operating characteristic curve, was 0.682 (95% CI, 0.618-0.746) for OS, which was higher than GGT and LMR. In conclusion, low preoperative LMR and high preoperative GGT were associated with a poor prognosis in HCC patients after hepatectomy. Our results confirmed that the SIS qualifies as a novel prognostic predictor of HCC patients after hepatectomy.

## INTRODUCTION

Liver cancer is one of the most common human malignancies and most primary liver cancers occurring worldwide are hepatocellular carcinoma (HCC) [1]. Surgery is the only potentially curative treatment option for patients who have resectable HCC. Unfortunately, even after surgery, the 5-year overall survival (OS) rate is estimated to 50% and the 5-year recurrence rate exceeds 70% [2, 3] .Thus, it is crucial to explore and identify biomarkers for predicting the prognosis in HCC patients after surgery.

Systemic inflammatory response is increasingly recognized to play decisive roles at different stages of tumor development, including initiation, promotion, malignant conversion, invasion, and metastasis [4]. Previous studies reported the systemic inflammatory response was associated with cancer progression [5]. Recently there has been increasing interest in improving cancer prognostication using inflammatory biomarkers. The neutrophil-to-lymphocyte ratio (NLR) and platelet-to-lymphocyte ratio (PLR), have both been demonstrated to be prognostic markers for patients with various types of tumors [6-14]. Previous studies of hematologic malignancies suggested that an increased LMR indicate a good prognosis [15, 16]. There have been a few reports focusing on the prognostic significance of LMR in patients with solid tumors, including gastric [17], colon[18], bladder[19,20], renal[21] and lung cancers [22].

Gamma-glutamyltransferase (GGT) plays an important role in the metabolism of glutathione. GGT was investigated as a liver enzyme and a high level of serum GGT has been usually deemed as an alert sign for potential liver disease clinically. Recently, some studies suggested elevated GGT was a promising biomarker for poor OS of HCC patients who underwent hepatic resection [23], radiofrequency-ablation treatment [24] or transcatheter arterial chemoembolization [25].

In this study, we created a systemic inflammation score (SIS) basing on preoperative serum GGT and LMR. It may serve as a better prognostic predictor for clinical outcome in HCC patients after hepatectomy. We conducted this retrospective study in a large cohort of HCC patients undergoing potentially curative resection, attempting to investigate the prognostic value of the systemic inflammatory biomarkers in HCC patients undergoing curative resection.

## RESULTS

### Clinicopathological characteristics

Clinical and pathologic characteristics of all patients were summarized in Table 1. Of the 271 patients, 213 (78.6%) were males and 58 (21.4%) were females. The median age of the entire cohort was 60 years (range, 27-81 years). The median follow-up was 26 months (range, 5 to 101 months). The mean preoperative GGT was 102.37±146.79 (U/L). The mean platelet, absolute lymphocyte and absolute monocyte counts were 188.36±64.92(×10^9^L^-1^), 1.61±0.56 (×10^9^L^-1^) and 0.45±0.20 (×10^9^L^-1^), respectively. The mean LMR was 4.09±1.99.

**Table 1:** Association of LMR,GGT and SIS with clinicopathological characteristics

	Total	LMR			GGT			SIS			
	*N*=271	<4.5 (*n*=170)	≥4.5 (*n*=101)	*P*	<50 (*n*=129)	≥50 (*n*=142)	*P*	0 (*n*=58)	1 (*n*=114)	2 (*n*=99)	*P*
Age(year)				0.157			0.909				0.602
≤50	106	61	45		50	56		26	43	37	
>50	165	109	56		79	86		32	71	62	
Sex				0.179			0.002				0.014
Female	58	32	26		38	20		19	26	13	
Male	213	138	75		91	122		39	88	86	
HBsAg				0.477			0.077				0.107
Positive	218	139	79		98	120		41	95	82	
Negative	53	31	22		31	22		17	19	17	
AFP				0.084			0.046				0.517
≤20μg/L	101	70	31		56	45		20	47	34	
>20μg/L	170	100	70		73	97		38	67	65	
TB				0.056			0.479				0.229
≤17.1umol/L	265	164	101		127	138		58	112	95	
>17.1umol/L	6	6	0		2	4		0	2	4	
Albumin				0.069			0.058				0.049
≤40 g/L	102	71	31		41	61		15	42	45	
>40 g/L	169	99	70		88	81		43	72	54	
ALT				0.393			0.001				0.007
≤40 U/L	221	136	85		116	105		51	99	71	
>40 U/L	50	34	16		13	37		7	15	28	
AST				0.064			<0.001				<0.001
≤40 U/L	230	139	91		121	109		54	104	72	
>40 U/L	41	31	10		8	33		4	10	27	
Liver cirrhosis				0.064			0.063				0.136
No	41	31	10		25	16		6	23	12	
Yes	230	139	91		104	126		52	91	87	
Tumor size				0.215			<0.001				0.001
≤5 cm	121	71	50		76	45		35	56	30	
>5 cm	150	99	51		53	97		23	58	69	
Tumor number				0.069			0.118				0.834
Single	207	136	71		104	103		45	85	77	
Mutiple	64	34	30		25	39		13	29	22	
Tumor encapsulation				0.937			0.018				0.181
Complete	135	85	50		74	61		31	62	42	
None	136	85	51		55	81		27	52	57	
Vascular invasion				0.903			0.131				0.647
No	181	114	67		92	89		41	77	63	
Yes	90	56	34		37	53		17	37	36	
Tumor differentiation				0.997			0.163				0.612
I+II	169	106	63		86	83		38	73	58	
III+IV	102	64	38		43	59		20	41	41	
TNM stage				0.020			<0.001				0.001
I	210	124	86		112	98		53	92	65	
II+III	61	46	15		17	44		5	22	34	

Abbreviations: AFP=alpha fetoprotein; ALT=alanine aminotransferase; AST=aspartate transaminase; GGT=gamma-glutamyltransferase; HBsAg=hepatitis B surface antigen; LMR= lymphocyte-to-monocyte ratio; SIS=systemic inflammation score; TB= total bilirubin; TNM = tumor–node–metastasis.

### The optimal cut-off value for LMR

The optimal cut-off value of LMR was determined by the receiver operating characteristic (ROC) curve analysis for OS. The cut-off value was 4.5 when OS was employed as end-point for LMR, which yielded the largest sensitivity and specificity. LMR was stratified into < 4.5 or ≥4.5 for subsequent analyses. 170 patients (62.7%) and 101 patients (37.3%) were included in the low-LMR group ( < 4.5) and high-LMR group (≥4.5), respectively.

### Systemic inflammation score (SIS) evaluation

The continuous variable GGT was stratified into < 50 or ≥50 U/L and the continuous variable LMR was stratified into < 4.5 or ≥4.5 for subsequent analyses. Kaplan-Meier analysis indicated that the high-GGT and low-LMR were both associated with shorter OS (*P* < 0.001 for both). In order to further discriminate patients with different outcome, we subsequently dichotomized patients into four subgroups basing on serum GGT and LMR levels. In subgroups of either high-GGT or low-LMR, the OS was not different significantly (*P* = 0.518). Therefore, these two subgroups were combined and SIS was scored as follows: Patients with low-GGT and high-LMR were allocated a score of 0, patients with either high-GGT or low-LMR were allocated a score of 1, and patients with both high-GGT and low-LMR were allocated a score of 2.

### Associations of LMR, GGT and SIS with clinicopathological characteristics

The clinicopathological characteristics grouped by LMR, GGT and SIS were summarized in Table 1. In patients with HCC, LMR was associated with TNM stage (*P* = 0.020). Elevated GGT was associated with male (*P* = 0.002), alpha fetoprotein(AFP)>20μg/L (*P* = 0.046), ALT>40U/L (*P* = 0.001), AST>40U/L (*P* < 0.001), tumor size >5 cm (*P* < 0.001), tumor encapsulation (*P* = 0.018) and TNM stage (*P* < 0.001). SIS was significantly associated with sex (*P* = 0.014), albumin (*P* = 0.049), ALT (*P* = 0.007), AST (*P* < 0.001), tumor size (*P* = 0.001) and TNM stage (*P* = 0.001).

### Analysis of the prognostic impact of LMR, GGT and SIS

**Figure 1:**
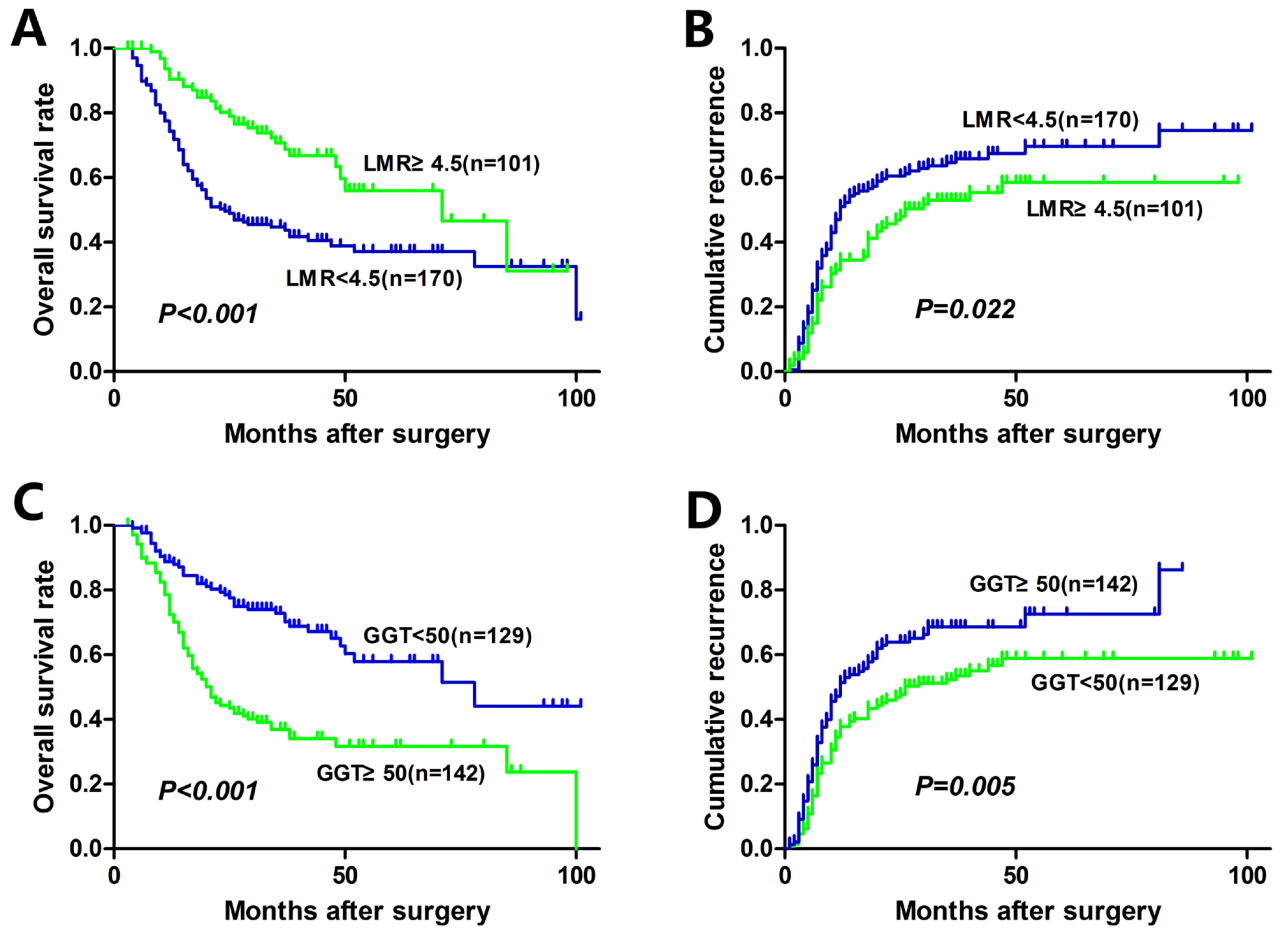
Kaplan-Meier analyses for overall survival and cumulative recurrence rate of HCC patients based on preoperative LMR and GGT

**Figure 2:**
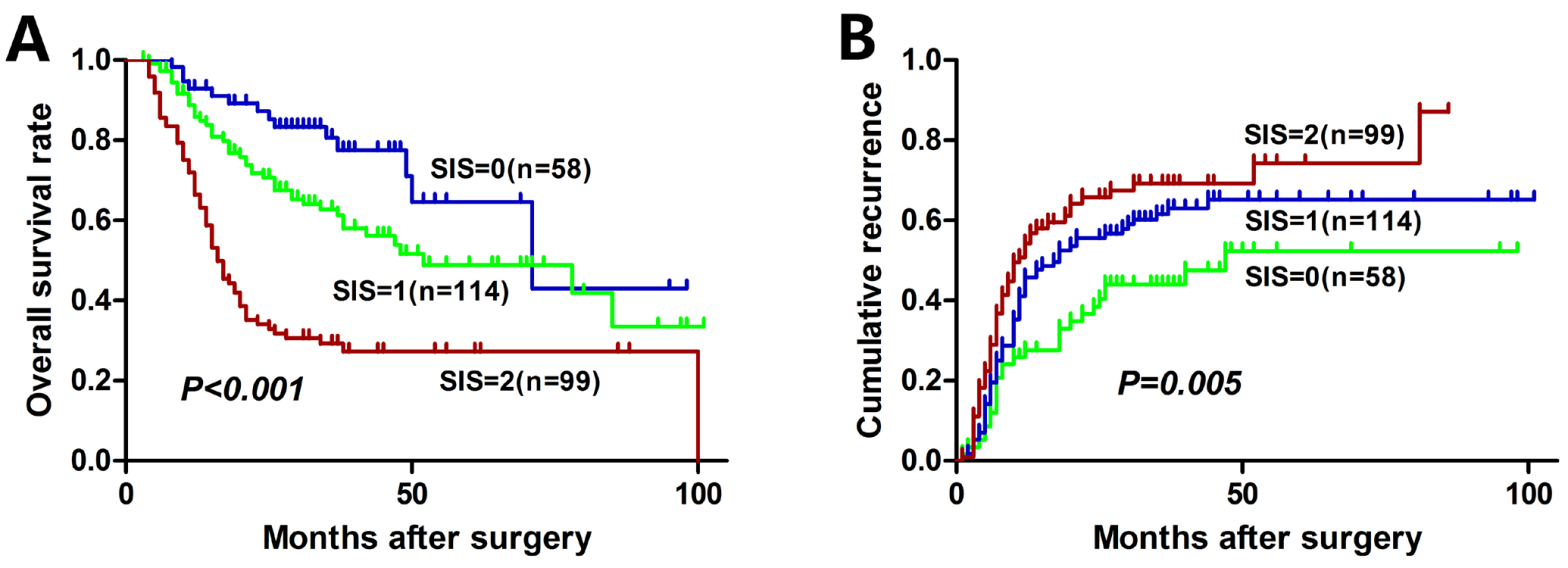
Kaplan-Meier analyses for overall survival and cumulative recurrence rate of HCC patients based on SIS

The median OS of the entire cohort was 29.3 months and 5-year OS was 36.6 %. The median time to recurrence (TTR) of the entire cohort was 18.0 months. The relationships between preoperative LMR, GGT, and OS and TTR were shown in Figgure 1. An elevated preoperative LMR was significantly associated with increased OS (*P* < 0.001, Figure 1A) and TTR (*P* = 0.022, Figure 1B). Whereas an elevated preoperative GGT was significantly associated with inferior OS (*P* < 0.001, Fig. 1C) and TTR (*P* = 0.005, Figure 1D). In addition, low SIS was significantly associated with increased OS (*P* < 0.001, Figure 2A) and TTR (*P* = 0.005, Figure 2B). The 1-, 3-, and 5-year OS rates of patients with SIS = 0 (89.5%, 65.9%, and 52.6%, respectively) were significantly higher than patients with SIS = 1 (76.3%, 52.2%, and 38.5%, respectively) and SIS = 2 (63.6%, 26.8%, and 25.1%, respectively). Moreover, the 1-, 3-, and 5-year cumulative recurrence rates of patients with SIS = 0 were 27.6%, 44.2%, and 52.7%, respectively, which were significantly lower than those of SIS = 1 (44.0%, 61.7%, and 65.4%, respectively) and SIS = 2 (53.3%, 69.5%, and 74.6%, respectively).

### Evaluation of the prognostic factors for OS and TTR using the Cox proportional hazard model

The results of univariate and multivariate Cox regression analyses of the factors related to OS and TTR were summarize in Table 2. Univariate analysis indicated that ALT, AST, tumor size, tumor encapsulation, vascular invasion, TNM stage, absolute lymphocyte counts, absolute monocyte counts, LMR, GGT and SIS were significant prognostic factors for OS, and tumor number, tumor encapsulation, vascular invasion, TNM stage, LMR, GGT and SIS were significant prognostic factors for TTR. Two multivariate models were performed separately, considering that SIS is constructed based on GGT and LMR. Multivariate analysis indicated that ALT, tumor encapsulation, vascular invasion, TNM stage, LMR, GGT and SIS were independent prognostic factors for OS, and tumor number, tumor encapsulation, vascular invasion, TNM stage, LMR and SIS were independent prognostic factors for TTR.

**Table 2:** Univariate and multivariate Cox proportional hazards regression analysis for OS and TTR

	OS		TTR	
	HR(95%CI)	*P*	HR(95%CI)	*P*
***Univariate analysis***				
Age ,year(>50y vs. ≤50)	1.110(0.775-1.589)	0.569	1.072(0.777-1.480)	0.672
Sex(male vs. female)	1.413(0.884-2.260)	0.148	1.305(0.866-1.965)	0.203
HBsAg(negative vs. positive)	0.853(0.546-1.331)	0.483	1.071(0.726-1.580)	0.729
AFP, μg/L(>20 vs. ≤20)	1.008(0.702-1.449)	0.964	1.094(0.786-1.523)	0.593
TB, umol/L(>17.1 vs. ≤17.1)	1.290(0.457-3.642)	0.631	0.491(0.121-1.982)	0.318
Albumin, g/L(>40 vs. ≤40)	0.772(0.538-1.107)	0.159	0.875(0.632-1.212)	0.421
ALT, U/L(>40 vs. ≤40)	1.713(1.139-2.576)	0.010	1.041(0.687-1.577)	0.852
AST, U/L(>40 vs. ≤40)	2.156(1.417-3.281)	<0.001	1.465(0.959-2.237)	0.077
Liver cirrhosis(yes vs. no)	1.316(0.788-2.198)	0.294	1.412(0.873-2.284)	0.159
Tumor size,cm(>5 vs. ≤5)	1.786(1.239-2.574)	0.002	1.366(0.992-1.881)	0.056
Tumor number (multiple vs. single)	1.278(0.846-1.929)	0.243	1.934(1.365-2.740)	<0.001
Tumor encapsulation (complete vs. none)	1.973(1.379-2.822)	<0.001	1.511(1.101-2.075)	0.011
Vascular invasion(yes vs. no)	2.174(1.530-3.090)	<0.001	1.707(1.237-2.356)	0.001
Tumor differentiation(III+IV vs I+II)	1.079(0.753-1.548)	0.678	1.283(0.931-1.770)	0.128
TNM stage (II+III vs. I)	2.252(1.525-3.328)	<0.001	1.880(1.311-2.697)	0.001
Absolute lymphocyte counts^1^	0.679(0.493-0.934)	0.017	0.866(0.652-1.150)	0.321
Absolute monocyte counts^1^	2.599(1.083-6.232)	0.032	1.931(0.876-4.257)	0.103
Absolute platelet counts^1^	1.001(0.999-0.004)	0.297	1.001(0.998-1.003)	0.636
LMR (≥4.5 vs. <4.5)	0.450(0.301-0.673)	<0.001	0.680(0.487-0.949)	0.023
GGT,U/L (≥50 vs. <50)	2.619(1.805-3.801)	<0.001	1.562(1.136-2.147)	0.006
SIS				
0	Reference		Reference	
1	1.913(1.051-3.481)	0.034	1.544(0.987-2.416)	0.057
2	4.695(2.631-8.378)	<0.001	2.124(1.348-3.347)	0.001
***Multivariate analysis*** ^2^				
ALT, U/L(>40 vs. ≤40)	1.995(1.100-3.618)	0.023	NA	
AST, U/L(>40 vs. ≤40)	0.899(0.501-1.615)	0.899	NA	
Tumor size,cm(>5 vs. ≤5)	0.972(0.601-1.572)	0.909	NA	
Tumor number (multiple vs. single)	NA		2.139(1.495-3.061)	<0.001
Tumor encapsulation (complete vs. none)	1.952(1.341-2.843)	<0.001	1.547(1.122-2.132)	0.008
Vascular invasion(yes vs. no)	2.026(1.329-3.089)	0.001	1.684(1.216-2.332)	0.002
TNM stage (II+III vs. I)	1.770(1.150-2.726)	0.010	1.589(1.097-2.303)	0.014
Absolute lymphocyte counts	0.807(0.534-1.222)	0.311	NA	
Absolute monocyte counts	0.600(0.178-2.024)	0.600	NA	
LMR (≥4.5 vs. <4.5)	0.467(0.273-0.800)	0.006	0.614(0.433-0.870)	0.006
GGT,U/L (≥50 vs. <50)	1.963(1.293-2.981)	0.002	1.228(0.886-1.702)	0.217
***Multivariate analysis*** ^3^				
ALT, U/L(>40 vs. ≤40)	1.963(1.085-3.552)	0.026	NA	
AST, U/L(>40 vs. ≤40)	0.885(0.494-1.586)	0.885	NA	
Tumor size,cm(>5 vs. ≤5)	0.956(0.598-1.528)	0.850	NA	
Tumor number (multiple vs. single)	NA		2.055(1.444-2.923)	<0.001
Tumor encapsulation (complete vs. none)	1.960(1.347-2.851)	<0.001	1.514(1.100-2.083)	0.011
Vascular invasion(yes vs. no)	2.056(1.348-3.136)	0.001	1.657(1.197-2.293)	0.002
TNM stage (II+III vs. I)	1.764(1.146-2.716)	0.010	1.585(1.094-2.295)	0.015
Absolute lymphocyte counts	0.791(0.542-1.155)	0.225	NA	
Absolute monocyte counts	0.611(0.192-1.949)	0.405	NA	
SIS				
0	Reference		Reference	
1	1.680(0.900-3.137)	0.103	1.460(0.930-2.291)	0.100
2	3.784(1.865-7.680)	<0.001	1.996(1.249-3.190)	0.004

Abbreviations: AFP=alpha fetoprotein; ALT=alanine aminotransferase; AST=aspartate transaminase; CI=confidence interval; GGT=gamma-glutamyltransferase; HBsAg=hepatitis B surface antigen; HR=hazard ratio; LMR= lymphocyte-to-monocyte ratio; NA=not adopted; OS= overall survival; SIS=systemic inflammation score; TB= total bilirubin; TNM stage= tumor–node–metastasis stage; TTR= time to recurrence.

^1^Analysed as a continuous variable.

^2^Analysis including LMR and GGT (omitting SIS).

^3^Analysis including SIS (omitting LMR and GGT).

### Comparation of predictive ability of SIS and other inflammatory parameters

**Figure 3:**
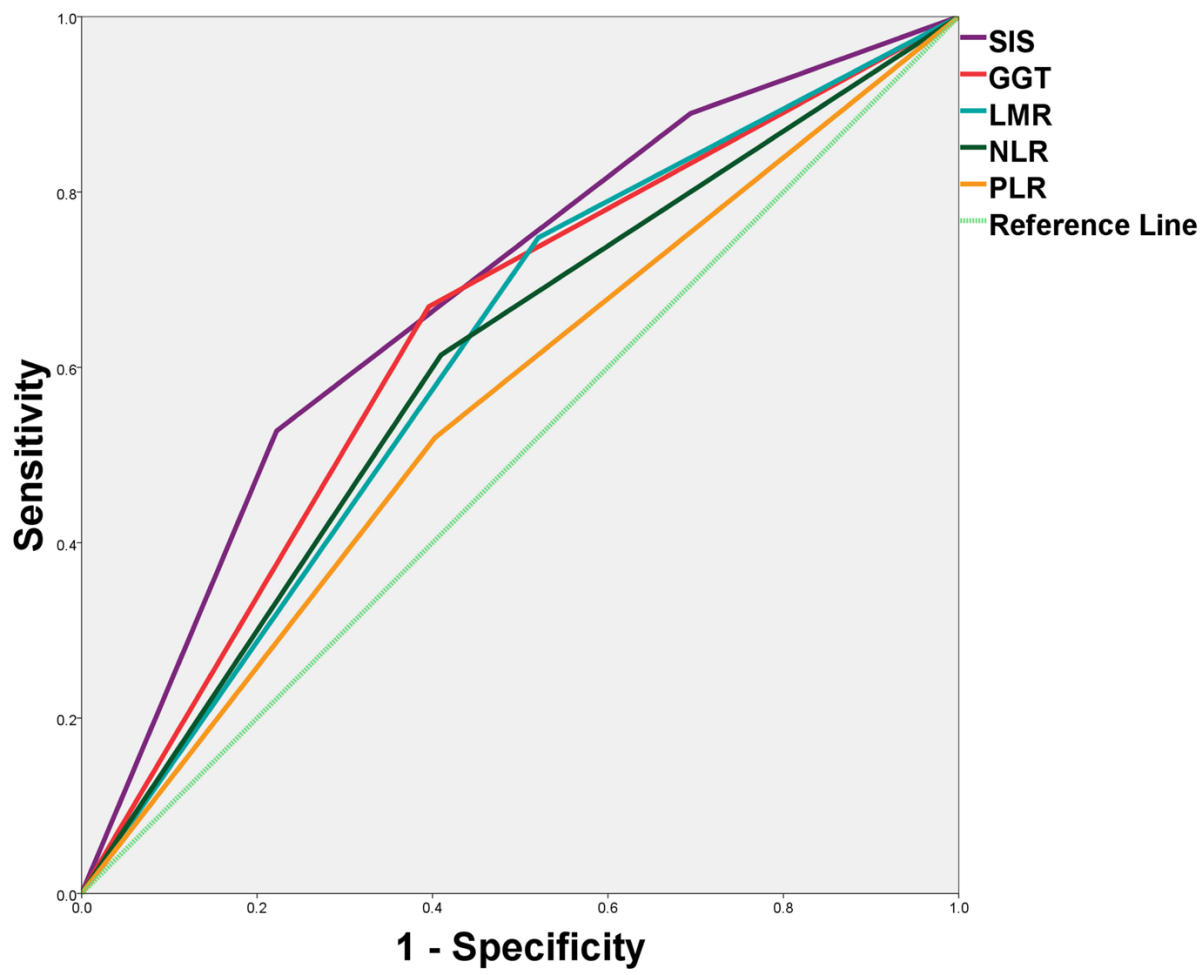
Predictive ability of the SIS was compared with other inflammatory parameters by ROC curves

Predictive ability of the SIS was compared with other inflammatory parameters(GGT, LMR, NLR and PLR) by ROC curves (Figure 3). The discrimination ability was compared by the area under the receiver operating characteristic curve(AUC) for OS (Table 3). The AUC for the SIS was 0.682 (95% CI, 0.618-0.746), which was the strongest factor among inflammatory parameters (GGT, LMR, NLR and PLR) for predicting survival in patients with HCC.

**Table 3:** Comparation of predictive ability of SIS and other inflammatory parameters

	AUC(95%CI)	*P*
SIS	0.682(0.618-0.746)	<0.001
GGT	0.637(0.570-0.703)	<0.001
LMR	0.614(0.547-0.681)	0.001
NLR	0.602(0.535-0.670)	0.004
PLR	0.558(0.490-0.627)	0.097

Abbreviations: AUC=area under the receiver operating characteristic curve; CI=confidence interval; GGT=gamma-glutamyltransferase; LMR=lymphocyte-to-monocyte ratio; NLR=neutrophil-to-lymphocyte ratio; PLR=platelet-to-lymphocyte ratio; SIS=systemic inflammation score.

## DISCUSSION

Links between cancer and inflammation were first described in the nineteenth century. Nowadays, increasing evidence indicating systemic inflammatory response plays an important role in cancer progression [5]. Markers based on systemic inflammation, such as the NLR and PLR, have been reported to be useful in predicting the outcome of cancer patients [6-13]. In the present study, a novel systemic inflammation score (SIS) was constructed based on preoperative serum GGT and LMR. The data indicated GGT, LMR and SIS were independent predictors of survival and recurrence for patients with HCC after hepatectomy. Our results revealed that an elevated preoperative LMR was significantly associated with increased OS and TTR, whereas an elevated preoperative GGT were significantly associated with inferior OS and TTR. The exact reason for the association of elevated preoperative GGT or low LMR with poor prognosis in malignant tumor patients remains largely unclear.

Firstly, our study identified GGT as a prognostic marker for patients with HCC after hepatectomy. GGT is a key enzyme that plays an important role in the metabolism of glutathione, and it is also correlated with tumorigenesis [26]. GGT may induced DNA instability and subsequent oncogenesis, leading to the death of normal liver cells or the loss of normal liver function [27]. A series of studies have suggested that serum GGT was a marker of oxidative stress [28]. The pro-oxidant activity of GGT may contribute to the persistent oxidative stress described in cancer and modulate processes involved in tumor progression [29]. As a consequence, recent studies suggested elevated GGT was a promising biomarker for poor OS of HCC patients who underwent hepatic resection [23], radiofrequency-ablation treatment [24] or transcatheter arterial chemoembolization [25]. The molecular mechanisms of the association between GGT and poor prognosis of HCC need further study.

The second part of the study was successful in defining the utility of the LMR as a prognostic indicator in HCC patients after hepatectomy. Lymphocytes can exert an anti-tumor effect by inhibiting tumor cell proliferation and migration [5,30]. As a consequence, a low lymphocyte count might result in a weak antitumor reactions and could predict a poor clinical outcome [31]. On the other hand, monocytes are a type of white blood cells that can further differentiate into a range of tissue macrophages and dendritic cells [32]. Monocytes were reported to promote tumorigenesis through local immune suppression [33]. In addition, monocytes can differentiate into tumor associated macrophages (TAMs), which mostly promote tumor growth and may be obligatory for angiogenesis, invasion, and metastasis [34]. Macrophage could also promote the growth, migration and metastasis of tumor cells by releasing some soluble factors [5, 35]. Previous studies indicated elevated macrophage content was associated with poor clinical outcome [36-38]. Hence, an elevated absolute monocyte count may predict poor prognosis in tumor patients. LMR, a combination of lymphocytes and monocytes, may represent a balance in host immunity against malignancy has enhanced prognostic value. Previous studies of hematologic malignancies suggested that an increased LMR promised a good prognosis [15,16]. More recently, some reports indicated the prognostic significance of LMR in patients with solid tumors, including gastric [17], colon [18], bladder [19, 20], renal [21] and lung cancers [22].

Our results indicated the predictive ability of GGT and LMR is stronger than other inflammatory parameters (NLR and PLR). Therefore, we combine these two prognostic markers to construct SIS, assuming that SIS might have a combined predictive effect of GGT and LMR. SIS is a convenient biomaker because serum GGT and complete blood count are routinely measured before surgery in our clinical practice. In the future, basic research may provide an understanding of its molecular mechanisms that may become potential therapeutic targets.

We assessed the association of LMR, GGT and SIS with clinicopathological characteristics. It is worth mentioning that GGT were significantly associated with male, ALT and AST in our study, which was in line with previous studies. Previous studies has demonstrated a sex difference in GGT level [39, 40] and it has been suggested that the lower level of GGT for women is likely to be of a physiological nature. Moreover, fatty liver occurred more frequently in men than women and the distributions of concentrations of liver enzymes differ between men and women [41]. Recommended cutoffs of abnormal liver enzymes were significantly higher for men than women [42].

The present study had several limitations that require discussion. Firstly, the present study was a retrospective design with single-center and missing variables or selection bias are possible because of its retrospective nature. Secondly, peripheral blood cell counts were performed only once, which might cause bias. In addition, C-reactive protein (CRP) was not gathered in our analyses because it was not routinely measured in our clinical practice. Large-scale prospective studies are warranted to substantiate and validate our results.

In conclusion, our results demonstrated low preoperative LMR and high preoperative GGT were associated with a poor prognosis in HCC patients after hepatectomy. SIS, constructed based on preoperative GGT and LMR, is an easily measured and novel prognostic marker that was significantly correlated with OS and TTR. Our results confirmed that the SIS qualifies as a novel prognostic predictor of HCC patients after curative resection.

## MATERIALS AND METHODS

### Patients

A total of 271 patients with HCC who underwent curative hepatic resection at the Liver Cancer Institute of Zhongshan Hospital (Fudan University, Shanghai, China) between 2008 and 2011 were enrolled after informed consent. Patients who underwent preoperative therapy, such as transarterial chemoembolization, radiofrequency ablation or percutaneous ethanol injection, were excluded from this study. Ethical approval was obtained from the research ethics committee of Zhongshan Hospital, and written informed consent was obtained from each patient.

### Follow-up

The patient follow-up and postoperative treatment were administrated as described previously according to our established guidelines [43]. All patients were regularly screened for recurrence through monitoring of serum AFP, abdomen ultrasonography, and chest x-ray every 1 to 6 months according to the postoperative time. For patients with test results suggestive of recurrence, computed tomography and/or magnetic resonance imaging were used to verify the recurrence. TTR was defined as the interval between the date of surgery and the first recurrence, or from the date of surgery to the date of last follow-up patients without recurrence. OS was defined as the interval between surgery and death, or the interval between surgery and the last observation for surviving patients. Patients who were still alive or recurrence-free were censored at the last follow-up date.

### Statistical analysis

Data are expressed as the mean ± standard deviation. ROC curve analysis was applied to determine the optimal cut-off level for LMR as predictor of OS. Prediction accuracy was evaluated with area under the ROC curve. The associations of LMR, GGT and SIS with clinicopathological characteristics were examined using the χ^2^ test or Fisher exact test. The Cox proportional hazards regression model was applied to perform univariate and multivariate analyses, and those variables that achieved statistical significance in the univariate analysis were entered into the multivariable analysis. The Kaplan-Meier method with log-rank test was used to compare survival curves. All statistical analyses were performed using the Statistical Package for Social Sciences version 19.0 (SPSS Inc, Chicago, IL). A two-sided *P*-value of < 0.05 was considered statistically significant in all tests.

## Abbreviations

AFP: alpha fetoprotein
ALT: alanine aminotransferase
AST: aspartate transaminase
AUC: area under the receiver operating characteristic curve
CI: confidence interval
GGT: gamma-glutamyltransferase
HBsAg: hepatitis B surface antigen
HCC: hepatocellular carcinoma
LMR: lymphocyte-to-monocyte ratio
NLR: neutrophil-to-lymphocyte ratio
OS: overall survival
PLR: platelet-to-lymphocyte ratio
ROC: Receiver operating characteristic
SIS: systemic inflammation score
TB: total bilirubin
TNM: tumor-node-metastasis
TTR: time to recurrence

## References

[1] Torre LA, Bray F, Siegel RL, Ferlay J, Lortet-Tieulent J, Jemal A (2015). Global cancer statistics, 2012. CA Cancer J Clin.

[2] Bruix J, Sherman M (2011). Management of hepatocellular carcinoma: an update. Hepatology.

[3] Grazi GL, Ercolani G, Pierangeli F, Del GM, Cescon M, Cavallari A, Mazziotti A (2001). Improved results of liver resection for hepatocellular carcinoma on cirrhosis give the procedure added value. Ann Surg.

[4] Grivennikov SI, Greten FR, Karin M (2010). Immunity, Inflammation, and Cancer. Cell.

[5] Mantovani A, Allavena P, Sica A, Balkwill F (2008). Cancer-related inflammation. Nature.

[6] Gomez D, Farid S, Malik HZ, Young AL, Toogood GJ, Lodge JP, Prasad KR (2008). Preoperative neutrophil-to-lymphocyte ratio as a prognostic predictor after curative resection for hepatocellular carcinoma. World J Surg.

[7] Cho H, Hur HW, Kim SW, Kim SH, Kim JH, Kim YT, Lee K (2009). Pre-treatment neutrophil to lymphocyte ratio is elevated in epithelial ovarian cancer and predicts survival after treatment. Cancer Immunol Immunother.

[8] Idowu OK, Ding Q, Taktak AF, Chandrasekar CR, Yin Q (2012). Clinical implication of pretreatment neutrophil to lymphocyte ratio in soft tissue sarcoma. Biomarkers.

[9] Kwon HC, Kim SH, Oh SY, Lee S, Lee JH, Choi HJ, Park KJ, Roh MS, Kim SG, Kim HJ, Lee JH (2012). Clinical significance of preoperative neutrophil-lymphocyte versus platelet-lymphocyte ratio in patients with operable colorectal cancer. Biomarkers.

[10] Raungkaewmanee S, Tangjitgamol S, Manusirivithaya S, Srijaipracharoen S, Thavaramara T (2012). Platelet to lymphocyte ratio as a prognostic factor for epithelial ovarian cancer. J Gynecol Oncol.

[11] Xue P, Kanai M, Mori Y, Nishimura T, Uza N, Kodama Y, Kawaguchi Y, Takaori K, Matsumoto S, Uemoto S, Chiba T (2014). Neutrophil-to-lymphocyte ratio for predicting palliative chemotherapy outcomes in advanced pancreatic cancer patients. Cancer Med.

[12] Chen Q, Dai Z, Yin D, Yang LX, Wang Z, Xiao YS, Fan J, Zhou J (2015). Negative impact of preoperative platelet-lymphocyte ratio on outcome after hepatic resection for intrahepatic cholangiocarcinoma. Medicine (Baltimore).

[13] Chen Q, Yang LX, Li XD, Yin D, Shi SM, Chen EB, Yu L, Zhou ZJ, Zhou SL, Shi YH, Fan J, Zhou J, Dai Z (2015). The elevated preoperative neutrophil-to-lymphocyte ratio predicts poor prognosis in intrahepatic cholangiocarcinoma patients undergoing hepatectomy. Tumour Biol.

[14] Lan H, Zhou L, Chi D, Zhou Q, Tang X, Zhu D, Yue J, Liu B (2017). Preoperative platelet to lymphocyte and neutrophil to lymphocyte ratios are independent prognostic factors for patients undergoing lung cancer radical surgery: A single institutional cohort study. Oncotarget.

[15] Li ZM, Huang JJ, Xia Y, Sun J, Huang Y, Wang Y, Zhu YJ, Li YJ, Zhao W, Wei WX, Lin TY, Huang HQ, Jiang WQ (2012). Blood lymphocyte-to-monocyte ratio identifies high-risk patients in diffuse large B-cell lymphoma treated with R-CHOP. Plos One.

[16] Porrata LF, Ristow K, Colgan JP, Habermann TM, Witzig TE, Inwards DJ, Ansell SM, Micallef IN, Johnston PB, Nowakowski GS, Thompson C, Markovic SN (2012). Peripheral blood lymphocyte/monocyte ratio at diagnosis and survival in classical Hodgkin’s lymphoma. Haematologica.

[17] Zhou X, Du Y, Xu J, Huang Z, Qiu T, Wang X, Qian J, Zhu W, Liu P (2014). The preoperative lymphocyte to monocyte ratio predicts clinical outcomes in patients with stage II/III gastric cancer. Tumour Biol.

[18] Shibutani M, Maeda K, Nagahara H, Ohtani H, Sakurai K, Yamazoe S, Kimura K, Toyokawa T, Amano R, Tanaka H, Muguruma K, Hirakawa K (2015). Prognostic significance of the lymphocyte-to-monocyte ratio in patients with metastatic colorectal cancer. World J Gastroenterol.

[19] Temraz S, Mukherji D, Farhat ZA, Nasr R, Charafeddine M, Shahait M, Wehbe MR, Ghaida RA, Gheida IA, Shamseddine A (2014). Preoperative lymphocyte-to-monocyte ratio predicts clinical outcome in patients undergoing radical cystectomy for transitional cell carcinoma of the bladder: a retrospective analysis. Bmc Urol.

[20] Zhang GM, Zhu Y, Luo L, Wan FN, Zhu YP, Sun LJ, Ye DW (2015). Preoperative lymphocyte-monocyte and platelet-lymphocyte ratios as predictors of overall survival in patients with bladder cancer undergoing radical cystectomy. Tumour Biol.

[21] Gu L, Ma X, Wang L, Li H, Chen L, Li X, Zhang Y, Xie Y, Zhang X (2017). Prognostic value of a systemic inflammatory response index in metastatic renal cell carcinoma and construction of a predictive model. Oncotarget.

[22] Hu P, Shen H, Wang G, Zhang P, Liu Q, Du J (2014). Prognostic significance of systemic inflammation-based lymphocyte- monocyte ratio in patients with lung cancer: based on a large cohort study. Plos One.

[23] Fu S, Guo Z, Li S, Kuang M, Hu W, Hua Y, He X, Peng B (2015). Prognostic value of preoperative serum gamma-glutamyltranspeptidase in patients with hepatocellular carcinoma after hepatectomy. Tumour Biol.

[24] Ma H, Zhang L, Tang B, Wang Y, Chen R, Zhang B, Chen Y, Ge N, Wang Y, Gan Y, Ye S, Ren Z (2014). gamma-Glutamyltranspeptidase is a prognostic marker of survival and recurrence in radiofrequency-ablation treatment of hepatocellular carcinoma. Ann Surg Oncol.

[25] Zhang JB, Chen Y, Zhang B, Xie X, Zhang L, Ge N, Ren Z, Ye SL (2011). Prognostic significance of serum gamma-glutamyl transferase in patients with intermediate hepatocellular carcinoma treated with transcatheter arterial chemoembolization. Eur J Gastroenterol Hepatol.

[26] Ikeda Y, Taniguchi N (2005). Gene expression of gamma-glutamyltranspeptidase. Methods Enzymol.

[27] Wang Z, Song P, Xia J, Inagaki Y, Tang W, Kokudo N (2014). Can gamma-glutamyl transferase levels contribute to a better prognosis for patients with hepatocellular carcinoma?. Drug Discov Ther.

[28] Lim JS, Yang JH, Chun BY, Kam S, Jacobs DJ, Lee DH (2004). Is serum gamma-glutamyltransferase inversely associated with serum antioxidants as a marker of oxidative stress?. Free Radic Biol Med.

[29] Corti A, Franzini M, Paolicchi A, Pompella A (2010). Gamma-glutamyltransferase of cancer cells at the crossroads of tumor progression, drug resistance and drug targeting. Anticancer Res.

[30] Rosenberg SA (2001). Progress in human tumour immunology and immunotherapy. Nature.

[31] Hoffmann TK, Dworacki G, Tsukihiro T, Meidenbauer N, Gooding W, Johnson JT, Whiteside TL (2002). Spontaneous apoptosis of circulating T lymphocytes in patients with head and neck cancer and its clinical importance. Clin Cancer Res.

[32] Auffray C, Sieweke MH, Geissmann F (2009). Blood monocytes: development, heterogeneity, and relationship with dendritic cells. Annu Rev Immunol.

[33] Gabrilovich DI, Nagaraj S (2009). Myeloid-derived suppressor cells as regulators of the immune system. Annu Rev Immuno.

[34] Condeelis J, Pollard JW (2006). Macrophages: obligate partners for tumor cell migration, invasion, and metastasis. Cell.

[35] Coussens LM, Werb Z (2002). Inflammation and cancer. Nature.

[36] Balkwill F (2004). Cancer and the chemokine network. Nat Rev Cancer.

[37] Tsutsui S, Yasuda K, Suzuki K, Tahara K, Higashi H, Era S (2005). Macrophage infiltration and its prognostic implications in breast cancer: the relationship with VEGF expression and microvessel density. Oncol Rep.

[38] Balkwill F, Charles KA, Mantovani A (2005). Smoldering and polarized inflammation in the initiation and promotion of malignant disease. Cancer Cell.

[39] Nilssen O, Forde OH, Brenn T (1990). The Tromso Study. Distribution and population determinants of gamma-glutamyltransferase. Am J Epidemio.

[40] Pintus F, Mascia P (1996). Distribution and population determinants of gamma-glutamyltransferase in a random sample of Sardinian inhabitants. ‘ATS-SARDEGNA’ Research Group. Eur J Epidemiol.

[41] Ford ES, Schulze MB, Bergmann MM, Thamer C, Joost HG, Boeing H (2008). Liver enzymes and incident diabetes: findings from the European Prospective Investigation into Cancer and Nutrition (EPIC)-Potsdam Study. Diabetes Care.

[42] Ruhl CE, Everhart JE (2009). Elevated serum alanine aminotransferase and gamma-glutamyltransferase and mortality in the United States population. Gastroenterology.

[43] Gao Q, Qiu SJ, Fan J, Zhou J, Wang XY, Xiao YS, Xu Y, Li YW, Tang ZY (2007). Intratumoral balance of regulatory and cytotoxic T cells is associated with prognosis of hepatocellular carcinoma after resection. J Clin Oncol.

